# Artificial Intelligence and FLIP Panometry—Automated Classification of Esophageal Motility Patterns

**DOI:** 10.3390/jcm15010401

**Published:** 2026-01-05

**Authors:** Miguel Mascarenhas, Francisco Mendes, João Rala Cordeiro, Joana Mota, Miguel Martins, Maria João Almeida, Catarina Araujo, Joana Frias, Pedro Cardoso, Ismael El Hajra, António Pinto da Costa, Virginia Matallana, Constanza Ciriza de Los Rios, João Ferreira, Miguel Mascarenhas Saraiva, Guilherme Macedo, Benjamin Niland, Cecilio Santander

**Affiliations:** 1 Gastroenterology Department, Centro Hospitalar Universitário São João, 4200-319 Porto, Portugal; 2 Faculdade de Medicina, Universidade do Porto, 4099-002 Porto, Portugal; 3 Department of Gastroenterology, University of South Alabama, Mobile, AL 36688, USA; 4 Telecomunications Institute, University Institute of Lisbon, 1499-066 Lisbon, Portugal; 5 Department of Information Science and Technology, University Institute of Lisbon, 1499-066 Lisbon, Portugal; 6 Department of Gastroenterology, Hospital Universitario Puerta de Hierro Majadahonda, 28222 Madrid, Spain; 7 Gastroenterology Department, Hospital Clinico San Carlos, 28040 Madrid, Spain; 8 Department of Mechanical Engineering, Faculdade de Engenharia, Universidade do Porto, 4099-002 Porto, Portugal; 9 ManopH Gastroenterology Clinic, 4000-432 Porto, Portugal; 10 Department of Gastroenterology, Hospital Universitario La Princesa, 28006 Madrid, Spain

**Keywords:** artificial intelligence, esophageal disorders, gastroenterology, machine learning, FLIP panometry

## Abstract

**Background/Objectives**: Functional lumen imaging probe (FLIP) panometry allows real-time assessment of the esophagogastric junction opening and esophageal body contractile activity during an endoscopic procedure. Despite the development of the Dallas Consensus, FLIP panometry analysis remains complex. Artificial intelligence (AI) models have proven their benefit in high-resolution esophageal manometry; however, data on their role in FLIP panometry are scarce. This study aims to develop an AI model for automatic classification of motility patterns during a FLIP panometry exam. **Methods**: A total of 105 exams from five centers from both the European and American continents were included. Several machine learning models were trained and evaluated for detection of FLIP panometry patterns. Each exam was classified with an expert consensus-based decision according to the Dallas Consensus, with division into a training and testing dataset in a patient-split design. Models’ performance was evaluated through their accuracy and area under the receiver-operating characteristic curve (AUC-ROC). **Results**: Pathological planimetry patterns were identified by an AdaBoost Classifier with 84.9% accuracy and a mean AUC-ROC of 0.92. Random Forest identified disorders of the esophagogastric junction opening with 86.7% accuracy and an AUC-ROC of 0.973. The Gradient Boosting Classifier identified disorders of the contractile response with 86.0% accuracy and an AUC-ROC of 0.933. **Conclusions**: In this study, integrating exams with different probe sizes and demographic contexts, a machine learning model accurately classified FLIP panometry exams according to the Dallas Consensus. AI-driven FLIP panometry could revolutionize the approach to this exam during an endoscopic procedure, optimizing exam accuracy, standardization, and accessibility, and transforming patient management.

## 1. Introduction

Esophageal motility disorders are common in clinical practice and impose a significant burden on both patients and the medical community. Frequently associated with a wide range of symptoms and disease presentations, their diagnosis is often time-consuming and may exacerbate disease morbidity. In this context, high-resolution esophageal manometry (HREM) is considered the gold standard for evaluating patients with suspected functional esophageal disorders, with the Chicago Protocol and Classification v4.0 guiding procedural performance and pattern recognition [1].

However, HREM is not universally available and requires complex interpretation. More importantly, it is often poorly tolerated, as it must be performed while the patient is awake. Large cohorts report a non-tolerability rate of around 2%, which can rise to 10% in children or in patients with prior upper gastrointestinal surgery [2]. Additionally, HREM presents several procedural limitations. Firstly, it offers a pressure-based assessment only, without evaluating esophagogastric junction (EGJ) distensibility, compliance, or diameter in real time. Secondly, it is highly dependent on patient cooperation, particularly due to the need for multiple repeated wet swallows. Finally, HREM has a recognized limitation in the evaluation of borderline EGJ disorders, in which distensibility metrics are often fundamental [3].

In 2014, functional lumen imaging probe (FLIP) panometry was introduced as a novel technique for the assessment of esophageal motility. Unlike high-resolution esophageal manometry (HREM), FLIP panometry performs impedance planimetry, providing measurements of cross-sectional luminal diameters, esophagogastric junction (EGJ) distensibility, and distension-induced contractile patterns (secondary peristalsis). While FLIP panometry demonstrates similar motility classification patterns to HREM in certain disorders, it offers important practical advantages: it is well tolerated under sedation and enables a “one-stop” diagnostic approach for patients with dysphagia, as it can be performed during the index sedated endoscopy [4].

Indeed, FLIP panometry has been demonstrated to have interesting applications in diverse esophageal motility disorders. Firstly, FLIP panometry has shown high correlation with HREM for diagnosis of achalasia, with detection of the cardinal feature of reduced EGJ opening [5]. Additionally, this procedure enables intra-operatory guidance of peroral endoscopic myotomy, leading to better patients outcomes with a decrease in post-procedure gastroesophageal reflux disease [6]. Considering EGJ outflow obstruction (EGJOO), FLIP panometry offers the possibility to clarify the clinical significance of suspected EGJOO per Chicago Classification v. 4.0 [7]. FLIP panometry findings are also good surrogate markers of symptom duration and diagnostic delay in eosinophilic esophagitis, being capable of identifying the cumulative fibrostenotic disease consequences [8]. Finally, this procedure has shown a high negative predictive value in case of a normal exam, suggesting a possible gastroesophageal reflux disease or functional syndrome as a cause of patient symptoms [9].

Nevertheless, several challenges remain before FLIP panometry can achieve widespread clinical implementation. First, it requires a standardized methodology for both procedure execution and motility pattern classification. In 2025, a panel of experts updated the Dallas Consensus, with uniformization of the FLIP panometry protocol, and interpretation of EGJ opening and contractile response, aiming to achieve a standardized approach for exam performance and analysis [9]. Second, there is ongoing debate regarding the potential effects of sedation on esophageal motility, although recent studies suggest that these effects are not clinically significant [10]. Third—and perhaps most importantly—FLIP panometry has a notable learning curve, and expertise in data interpretation is still limited, particularly for subtle abnormalities in EGJ mechanics or contractile responses. This results in significant variability between high-volume referral centers and low-resource settings, where the technique could be especially valuable. Combined with the high cost of the device and disposable catheters, these factors currently limit the generalization and routine adoption of FLIP panometry in clinical practice.

Over the last decade, there has been exponential growth in the development of artificial intelligence (AI) tools for gastroenterology, largely driven by the strong imaging-based nature of the field [11]. In this context, AI has demonstrated clinical benefit in several areas, including capsule endoscopy, upper gastrointestinal endoscopy, and colonoscopy [12,13]. Regarding esophageal motility disorders, a limited number of studies have evaluated AI-based identification of motility patterns in high-resolution esophageal manometry (HREM) [14,15]. In contrast, for FLIP panometry, only a single study to date has highlighted the potential of AI-assisted analysis to differentiate normal from pathological motility patterns, but there remains a lack of AI models integrated with the Dallas Consensus classification framework [16]. The present study aims to develop and validate an AI model capable of automatically classifying esophageal motility patterns during FLIP panometry examinations, based on the standardized Dallas Consensus criteria.

## 2. Materials and Methods

**Figure 1:**
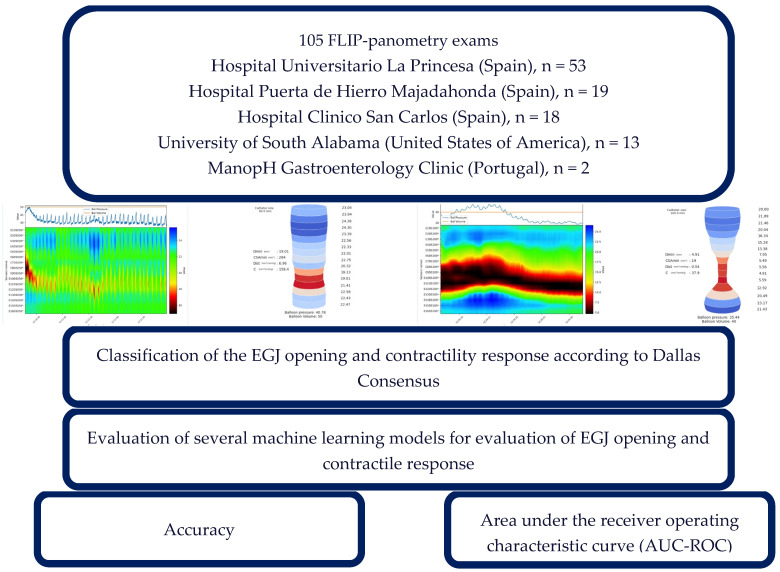
Study design and labelling according to Dallas Consensus. EGJ—esophagogastric junction opening.

A total of 105 FLIP panometry examinations from five reference centers—three in Spain (Hospital Universitario La Princesa, Hospital Clínico San Carlos, and Hospital Universitario Puerta de Hierro Majadahonda), one in the United States of America (University of South Alabama), and one in Portugal (ManopH Gastroenterology Clinic)—were included for model development (Figure 1). The dataset comprised procedures performed with both 8 cm and 16 cm EndoFLIP™ (Medtronic, Minneapolis, MN, USA) measurement catheters. All procedures were independently reviewed and labeled by two expert gastroenterologists according to the Dallas Consensus classification. In cases of disagreement, a consensus was reached through joint review; examinations for which consensus could not be achieved were excluded. Each case was annotated, classifying motility in two main domains (Figure 1):

- EGJ opening: normal, reduced, or inconclusive findings.
- Contractile response (CR): spastic, normal, diminished, absent, or inconclusive CR.

This study was conducted in accordance with the Declaration of Helsinki and was non-interventional in nature. Ethical approval was obtained from the institutional review board (IRB) or equivalent ethics committee at each participating center. All potentially identifying patient information was removed, and a random identifier was assigned to each examination to ensure complete data anonymization. A certified Data Protection Officer (DPO) supervised data handling to guarantee compliance with the General Data Protection Regulation (GDPR).

### 2.1. FLIP Panometry Procedure

The majority of FLIP procedures were performed before the publication of version 2.0 of the Dallas Consensus, explaining the variability in procedure performance. Procedures were performed with the 16 cm (70 exams) and 8 cm (35 exams) EndoFLIP™ catheters. Patients fasted for at least 6 h before the procedure. After performance of an upper endoscopy and measurement of EGJ location, sequential FLIP filling was achieved at least to 50 mL (in some cases, up to 70 mL, as recommended per protocol), with evaluation of EGJ parameters and CR in segments of at least 30 s of stabilized volume. The EGJ-distensibility index was achieved at 60 mL wherever possible, whereas the maximum EGJ diameter was determined at the largest filled volume. CR was evaluated mainly with the 16 cm catheters (given the lack of representation of esophageal motility with the 8 cm catheters).

This study focused on developing a model capable of detecting disorders of EGJ and CR in FLIP panometry studies according to the Dallas Consensus. Reduced EGJ opening is defined as a maximum EGJ diameter under 12 mm with an EGJ-distensibility index under 2.0 mm^2^/mmHg at 60 mL. Normal EGJ opening was defined as an EGJ diameter above 16 mm with an EGJ-distensibility index above 2.0 mm^2^/mmHg at 60 mL. An EGJ opening was labelled as inconclusive in any case not meeting criteria for normal or reduced EGJ opening. Regarding the CR, a pathological CR was defined as any case of spastic contraction (sustained lower esophageal sphincter contractions or sustained occluding contractions), reduced contraction, or absent contractions.

### 2.2. Model Selection and Tuning

The objective of this study was to identify a machine learning (ML) model capable of detecting pathological FLIP panometry exams, and to further classify disorders of EGJ opening and disorders of CR. To achieve this, multiple ML classifiers were trained and compared to determine the optimal model for our dataset. ML models were trained with time-series data from selected volumes (according to the considered volumes for EGJ distensibility and CR in Dallas Consensus). An inconclusive classification resulted in exclusion of the case from the training and testing sets for the specific parameter to which the inconclusive label applied. For the evaluation of the planimetry pattern and EGJ opening, the data were split into training (80%) and testing (20%) sets using stratified sampling to preserve class distribution. Given the higher percentage of inconclusive exams for the evaluation of CR (due to approximately one-third of the exams being performed with the 8 cm catheter), the data for the evaluation of CR disorders were split into training, validation, and testing sets with a 70%/15%/15% ratio. A broad range of classifiers was evaluated, including the XGB Classifier, LGBM Classifier, AdaBoost Classifier, GaussianNB, BernoulliNB, Bagging Classifier, Perceptron, Decision Tree Classifier, Extra Trees Classifier, Random Forest Classifier, Logistic Regression, SGD Classifier, Nearest Centroid, Extra Tree Classifier, SVC, Passive Aggressive Classifier, Linear SVC, K Neighbors Classifier, Linear Discriminant Analysis, Ridge Classifier CV, Ridge Classifier, Quadratic Discriminant Analysis, Label Spreading, Label Propagation, and Calibrated Classifier CV. A Dummy Classifier was included as a baseline for comparison. To ensure robust model selection, the dataset was split and models were trained in 10 independent iterations of the training dataset, and the top five models were selected based on weighted F1-score and area under the receiver operating characteristic curve (AUC-ROC). The three best-performing models underwent hyperparameter optimization using a genetic algorithm, and the best-tuned architecture was then re-trained five times to assess reproducibility and finalize the classifier. The best models were evaluated with an independent testing dataset. All analyses were performed on a workstation equipped with a 2.1 GHz Intel Xeon Gold 6130 processor (Intel, Santa Clara, CA, USA) and dual NVIDIA Quadro RTX 8000 graphics processing units (NVIDIA, Santa Clara, CA, USA).

### 2.3. Statistical Analysis

The performance of the ML models was assessed based on their accuracy and area under the receiver operating characteristic curve (AUC-ROC) for the detection of pathological FLIP panometry exams, disorders of EGJ opening, and disorders of CR. Model outputs were compared against the gold standard established by expert consensus.

## 3. Results

### 3.1. Study Population

**Figure 2:**
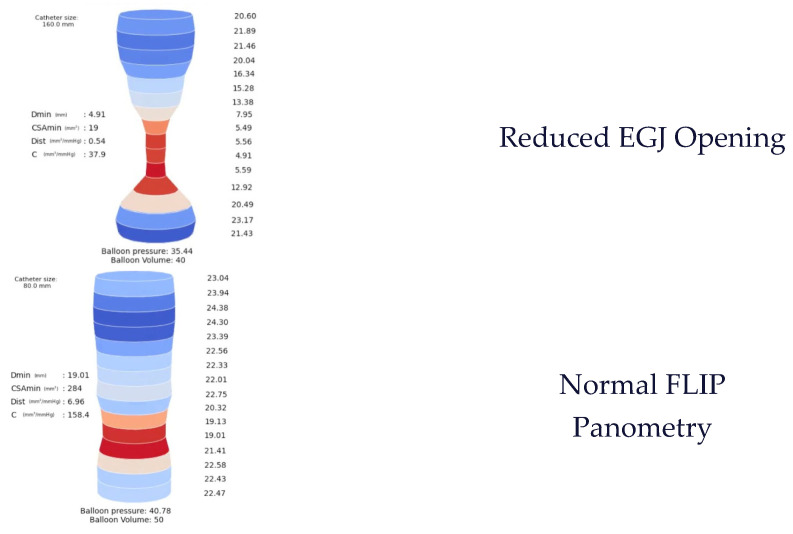
Examples of EGJ opening patterns in FLIP panometry in patient study population. EGJ—esophagogastric junction.

A total of 105 FLIP panometry examinations were included for model development and testing. A total of 60 patients presented with pathological findings (involving EGJ opening and/or CR), 15 patients had a normal exam, and 29 patients had at least one inconclusive finding (either in EGJ opening or CR evaluation). Regarding EGJ opening, 46 exams were classified as normal, 50 as pathological, and 9 as inconclusive. For contractile response (CR), 29 exams were normal, 29 pathological, and 47 inconclusive—the latter mainly related to the use of the 8 cm catheter, which limits assessment of complete esophageal contractility. Figure 2 provides examples of representative FLIP panometry patterns (specifically regarding EGJ opening) observed in the study population.

### 3.2. Model Performance

**Figure 3:**
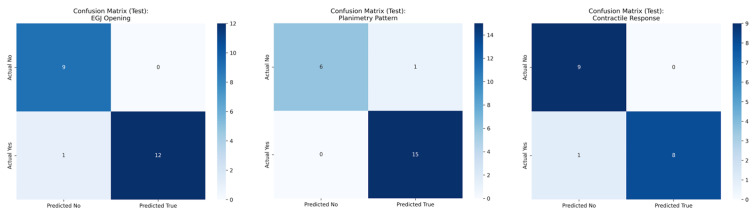
Confusion matrix of the testing dataset for detection of pathological planimetry patterns, disorders of esophagogastric junction opening, and contractile response in FLIP panometry exams.

Several ML models were evaluated for the automatic classification of esophageal motility patterns during FLIP panometry examinations, following the standardized Dallas Consensus criteria, performing significantly better than the Dummy Classifier. Table 1 summarizes the agreement between predictions from the most accurate ML models and the expert-labeled reference diagnoses (mean of 10 iterations within the testing dataset). Figure 3 showcases confusion matrixes for pathological planimetry patterns, disorders of EGJ opening and CR. The AdaBoost Classifier achieved an accuracy of 84.9% for detecting pathological planimetry patterns, with a mean AUC-ROC of 0.892. EGJ opening was most accurately classified using the Random Forest Classifier, which reached an accuracy of 86.7% and an AUC-ROC of 0.973. Finally, Gradient Boosting provided the best performance for CR classification, with a mean accuracy of 86.0% and an AUC-ROC of 0.933.

## 4. Discussion

The application of artificial intelligence (AI) models in medicine has experienced exponential growth, and gastroenterology is no exception. However, in the field of esophageal motility, we remain in the early stages of AI application. In this pilot study, which integrates data from both European and American populations, the authors present the first investigation of ML models for the automatic classification of FLIP panometry examinations with EndoFLIP™ according to the Dallas Consensus, evaluating both EGJ opening and contractile response. The application of such models could enhance generalizability in low-resource settings, enabling a single-step evaluation of patients with symptoms suggestive of esophageal motility disorders, performed during the index endoscopy.

In fact, FLIP panometry has undergone progressive evolution in both its clinical indications and the metrics available for interpretation [17,18]. Initially developed for the evaluation of esophageal motility disorders, particularly achalasia, its use has expanded in recent years to include applications in eosinophilic esophagitis and even gastroesophageal reflux disease (GERD) [8,19,20]. Additionally, FLIP panometry has demonstrated promising value in the periprocedural assessment of patients with achalasia undergoing peroral endoscopic myotomy (POEM) or endoscopic balloon dilation [21]. Nevertheless, several challenges remain that hinder the widespread adoption of this technology. First, FLIP panometry remains a relatively costly procedure, with recent analyses estimating an average cost of USD 568 per exam [22]. However, the same study acknowledges the potential cost-effectiveness of FLIP when considering the broader societal and clinical costs of a missed diagnosis. More importantly, despite recent efforts to standardize procedural performance and interpretation criteria, FLIP panometry still presents a notable learning curve. While excellent inter-observer agreement has been reported among experts in referral centers, there is growing concern about the risk of high inter-observer variability in non-specialist settings—particularly in the recognition of key motility features such as repetitive antegrade contractions (RACs) and subtle changes in EGJ distensibility index [4].

Considering FLIP-panometry’s intrinsic challenges, AI may offer a solution to increase diagnostic accuracy and reduce inter-observer variability, allowing for exam performance associated with index endoscopy, even in low-volume, less experienced centers. However, studies about AI implementation in FLIP panometry are scarce and require further validation. Recently, a study cohort of 678 patients and 35 asymptomatic controls resulted in a two-stage supervised deep learning model for analysis of FLIP panometry heatmaps generated from raw data, discriminating between normal and abnormal cases, and subdividing the latter into achalasia and non-achalasia phenotypes [16]. Despite the global accuracy of 89% in a large patient dataset, the model lacked validation using Dallas Consensus-based labels, but it represented a first step for AI-driven FLIP panometry analysis. Recently, a Python-based framework (MechView Program) was developed to standardize FLIP panometry measurements, providing automatic outputs to the clinician and helping achieve a faster panometry-driven diagnosis [23]. Despite being interesting, this framework only works as a semi-automated program, facilitating the automatic identification and calculation of fundamental metrics. However, clinical interpretation and analysis remain a main concern, as in low-resource settings, low diagnostic accuracy may be observed—particularly in more subtle cases of altered EGJ or CR. This study addresses some of the limitations of previous work, allowing for an automatic classification of FLIP panometry in compliance with the Dallas Consensus, showcasing a framework aligned with the current standard of care, and supporting the development of a possible AI-driven FLIP panometry model that enables broader implementation in low-volume centers—potentially modifying the diagnostic workup of patients with non-structural dysphagia.

Regarding the current challenges of FLIP panometry interpretation, the latest version of the Dallas Consensus highlighted the importance of recognizing subtle alterations in both EGJ opening and contractile response (CR) as surrogates of disorders of EGJ outflow [9]. Nevertheless, significant challenges remain due to device-related limitations: currently available 8 cm catheters are primarily designed to evaluate EGJ opening, whereas CR assessment is typically more accurate with 16 cm catheters [24]. Our dataset included exams performed with both catheter types, which may partly explain the higher proportion of inconclusive CR evaluations. Nevertheless, the catheter length heterogeneity should also be understood as a proof of model interoperability. In fact, this is one of the main principles for AI technology development and application, justified by the need of developing a system suitable for incorporation into every available device [25,26]. In this context, using both an 8 and 16 cm catheter is fundamental to address this interoperability challenge, creating a model with a higher technology readiness level.

Another important challenge addressed in the Dallas 2.0 Consensus is the interpretation of inconclusive EGJ opening, which may represent a surrogate for esophageal functional disorders [27]. Given the early technology readiness level of this work, the authors focused on developing binary classifications for both EGJ opening and CR. Consequently, inconclusive EGJ opening cases were not included in model training or testing, representing a limitation for model validation. Finally, there is significant heterogeneity between cases in terms of catheter maximum filling volumes, justified by the absence of the newly created Dallas Consensus. This problem was partially addressed by ML model training with time-series data from Consensus-recommended volumes for each specific metric, aiming to replicate the analysis in clinical practice according to the most recent evidence. Nevertheless, future studies should train and validate ML models with predefined maximum volumes and filling protocols. Additionally, future works will aim to validate a model capable not only of detection but also of distinguishing motility patterns in FLIP panometry exams, including the integration and classification of inconclusive cases, based on a standardized Dallas protocol.

On the other hand, consideration must be made regarding the impact of demographic context for the creation of a balanced dataset, fundamental for technology implementation across different clinical contexts. In fact, demographic bias has been shown to cause imbalances in AI model development, with an associated risk of inaccurate predictions [25,28]. In order to address this factor, this study was designed to incorporate data from five reference centers across three countries, assuring the inclusion of patients from different demographic contexts, with technology development and testing across both European and American populations.

Despite the promising results, FLIP panometry remains a complex procedure to interpret, relying heavily on clinician expertise. In the context of AI implementation in the motility field, the black-box nature of ML models, combined with exam subjectivity, may hinder a clear understanding of the rationale behind the model’s decisions, reducing clinician confidence in the technology [29]. Therefore, future research should focus on the development of explainable AI (XAI) tools, which can enhance clinician trust in the decision-making process [30,31,32]. The integration of such mechanisms with standardized diagnostic frameworks, such as the Dallas Consensus, would support the implementation of AI-driven FLIP panometry, improving both the trustworthiness and reliability of the models.

Considering the study’s results, some limitations must be acknowledged. Firstly, the study was based on a small dataset, albeit with demographic variability, which limits the technology’s applicability in clinical practice. Additionally, there was a significant discrepancy between the centers’ contributions, and a small testing dataset led to a wide distribution of accuracy values. Our group is currently focused on augmenting the dataset, which is fundamental for the development of accurate ML models. Secondly, the exclusion of examinations in which expert consensus was not achieved may reduce the number of challenging cases available for analysis, potentially limiting model performance in such scenarios. Moreover, the model used a binary classification for each parameter (normal vs. abnormal), without distinguishing between different types of disorders. Additionally, the “inconclusive” EGJ opening category was not incorporated into this pilot study, thereby reducing the system’s ability to recognize subtle alterations in EGJ opening. In this context, future studies should aim not only to identify EGJ opening and CR abnormalities, but also to automatically detect inconclusive EGJ openings and differentiate among CR patterns. Furthermore, future work will focus on developing a multimodal model for the evaluation of esophageal disorders, integrating FLIP panometry metrics with patient-reported symptoms and complementary diagnostic modalities such as HREM.

## 5. Conclusions

In conclusion, this study is the first worldwide to develop and validate an ML-based AI model for classification of FLIP panometry exams based on the Dallas Consensus. By incorporating data from both the European and American continents, the present study accurately identified disorders of EGJ opening and CR in diverse clinical settings. An AI-driven approach could fully leverage the potential of FLIP panometry when performed during index endoscopy, revolutionizing the management of patients with esophageal motility disorders.

## Figures and Tables

**Table 1:** Accuracy of the best machine learning model after fine-tuning for classification of motility patterns in FLIP panometry exams. SD—standard deviation. EGJ—esophagogastric junction. Accuracy evaluation represents a mean of 10 iterations within the testing dataset.

Parameter	Model	Accuracy, % Mean (SD)	ROC AUC Mean (SD)
Planimetry Pattern	AdaBoost Classifier	84.9 (8.2)	0.892 (0.060)
EGJ Opening	Random Forest	86.7 (9.6)	0.973 (0.029)
Contractile Response	Gradient Boosting	86.0 (7.3)	0.933 (0.067)

## Data Availability

Additional data are available upon reasonable request.
